# Two-Dimensional Films Based on Graphene/Li_4_Ti_5_O_12_ and Carbon Nanotube/Li_4_Ti_5_O_12_ Nanocomposites as a Prospective Material for Lithium-Ion Batteries: Insight from Ab Initio Modeling

**DOI:** 10.3390/ma16083270

**Published:** 2023-04-21

**Authors:** Vladislav V. Shunaev, Alexander A. Petrunin, Haifei Zhan, Olga E. Glukhova

**Affiliations:** 1 Department of Physics, Saratov State University, 410012 Saratov, Russia; sacha.petrynin@gmail.com (A.A.P.); glukhovaoe@sgu.ru (O.E.G.); 2 College of Civil Engineering and Architecture, Zhejiang University, Hangzhou 310058, China; zhan_haifei@zju.edu.cn

**Keywords:** Li_4_Ti_5_O_12_, graphene, carbon nanotubes, ab initio, density of states, quantum capacitance, li-ion batteries

## Abstract

The combination of spinel Li_4_Ti_5_O_12_ (LTO) with carbon nanostructures, such as graphene (G) and carbon nanotubes (CNTs), provides all of the required properties for modern chemical power sources such as Li-ion batteries (LIBs) and supercapacitors (SCs). G/LTO and CNT/LTO composites demonstrate a superior reversible capacity, cycling stability, and good rate performances. In this paper, an ab initio attempt to estimate the electronic and capacitive properties of such composites was made for the first time. It was found that the interaction between LTO particles and CNTs was higher than that with graphene due to the larger amount of transfer charge. Increasing the graphene concentration raised the Fermi level and enhanced the conductive properties of G/LTO composites. For CNT/LTO samples, the radius of CNT did not affect the Fermi level. For both G/LTO and CNT/LTO composites, an increase in the carbon ratio resulted in a similar reduction in quantum capacitance (QC). It was observed that during the charge cycle in the real experiment, the non-Faradaic process prevailed during the charge cycle, while the Faradaic process prevailed during the discharge cycle. The obtained results confirm and explain the experimental data and improve the understanding of the processes occurring in G/LTO and CNT/LTO composites for their usages in LIBs and SCs.

## 1. Introduction

Chemical power sources have become essential components of modern portable electronic devices, electric vehicles, and rechargeable electrochemical energy storage. For example, Li-ion batteries (LIBs) display a high energy density as well as long service life, low cost, and environmental friendliness. The search and synthesis of new materials that can improve the properties of LIBs is one of the main challenges nowadays. Spinel Li_4_Ti_5_O_12_ (LTO) has been widely applied as an anode material for LIBs and supercapacitors (SCs) due to its near-zero strain, thermal stability, good Li-ion intercalation, deintercalation reversibility, and average storage voltage, which forbids the formation of solid electrolyte interphase (SEI) films and lithium dendrite [1,2,3,4]. The application of LTO is limited by its extremely low conductivity (<10^−13^ S/cm), sluggish Li^+^ diffusion coefficient (<10^−8^ cm^2^/s), and poor theoretical capacity (~175 mAh/g) [5,6,7,8]. These drawbacks lead to a poor high-rate charge–discharge capability, large resistivity, and significant reduction in initial capacity after many cycles. To overcome these shortcomings, several attempts at LTO integration with superconductive and mechanically stable carbonaceous materials, such as carbon nanotubes (CNT) and graphene (G), have been made [9,10,11,12,13,14,15,16,17,18,19]. Thus, crystalline structures capable of reversible electrode oxidation or reduction at a high rate are in demand. Carbon nanostructures are functional and provide fast reversible electrode reactions which are the basis for the functioning of batteries capable of rapid energy exchange. Electrochemical systems based on carbon nanostructures decorated with electrochemically active materials can combine the advantages of two types of current sources.

Yan et al. synthesized Li_4_Ti_5_O_12_/graphene (LTO/G) nanocomposite in situ, in which graphene provided a conductive network for enhanced electron transport, while LTO grains ensured Li-ion pathways [9]. The obtained hybrid electrode demonstrated a high reversible capacity, good rate performance, and excellent cycling stability. Ding et al. reported that LTO/G prepared by the hydrothermal method showed a stronger pseudocapacitive effect during the discharge–charge processes as a result of the presence of LTO and a decreased transfer resistance as a result of the presence of graphene [10]. Introducing wrinkled graphene layers between the active spinel LTO and aluminum current collector using a blade coating method significantly improved the LTO material’s rate performance and cycling stability, as graphene provides a larger contact area, lower contact resistance, and stronger adhesion between the active material and the current collector [11]. Chen et al. developed a method for the controllable synthesis of large-scale 2D graphene-based LTO nanosheets with a hierarchical pore structure using amorphous TiO_2_ decorated reduced graphene oxide (rGO) as the precursor [12]. It was shown that the hierarchical structured LTO that was obtained exhibited a superior reversible capacity and cycling stability at high rates (e.g., 168 mAh/g at 10 °C after 1000 cycles). Zhang et al. [13] reported the excellent electrochemical performance of LTO synthesized by interfacial electrostatic self-assembly in a water-in-oil microemulsion system. They believed that the uniformly dispersed nanosized LTO particles are tightly anchored on graphene, which would shorten the lithium-ion migration path, expose more active sites, and improve the conductivity. Lin et al. obtained flower-like LTO hollow microspheres wrapped in graphene sheets through electrostatic interactions [14]. Based on energy-band model, they explained that the improved performance was connected to reduced work function.

Kim et al. synthesized 10–15 nm sized LTO nanoparticles uniformly decorated on CNTs using a microwave-solvothermal reaction [15]. The nanocomposite displayed a high-rate performance due to the shorter diffusion length of the LTO. The in situ growth of CNTs on the surface of LTO delivered superior reversible charge capacities (149.2, 102.6, 73.3, and 47.5 mAh/g at 0.2 °C, 10 °C, 20 °C, and 50 °C) in comparison with pure LTO [16]. Fabricated by the sol–gel method combined with a low temperature reflux process and a short post annealing, the carbon nanotube/Li_4_Ti_5_O_12_ (CNT/LTO) exhibited outstanding conductivity and rapid transfer due to their unique nanocable structure and hierarchical pores [17]. Wang et al. reported that well-dispersed CNTs in the matrix constructed conducting bridges between the LTO particles and enhanced the electron transport of the whole electrode [18]. Derived through the sol–gel process, CNT/LTO demonstrated an initial capacity (~140 mAh/g) retention of 87.8% after 2200 cycles at 1 A/g when the concentration of CNTs was equal to 11% and the LTO nanoparticles were connected by uniformly dispersed CNTs [19].

It should be noted that several attempts at exploring the properties of LTO at an atomic level have been made via molecular modeling. The calculations show that the energy band of LTO lies in the range of 1.7–2.3 eV, while in experiments, the energy band varies from 1.8 to 3.8 eV [20]. Through the PW91 + GGA approximation, Ouyang et al. found that band gap in LTO was opened between the occupied oxygen p-states and the empty Ti d-states [21]. The influence of metal doping on LTO conductivity was estimated and it was established that Mg-doped LTO could be considered as the most successful material for application in LIBs and SCs. It was also concluded that the mechanism of electron transport depends on the hopping behavior of Ti^4+^-Ti^3+^ [22]. Liu et al. revealed that optical properties of LTO in the infrared and the visible region were derived from the electronic transition between O 2p and Ti 3d states near the Fermi level, while optical properties in the ultraviolet region depended on the transition from O 2p valence band states to Ti 3d conduction band states [23].

Despite the many experimental works devoted to the application of G/LTO and CNT/LTO in chemical power sources, there have been no attempts made to estimate its electronic and energetic properties via molecular modeling tools. Some novel theoretical data on the process of the interaction between carbon materials and LTO (for example, formation energy for different mass ratios) could improve the existing methods of composite synthesis and save time for the developers of LIBs and SCs on its base. In this paper, using ab initio methods, we will build G/LTO and CNT/LTO supercells with different mass ratios of LTO for the first time. For these supercells, DOS and pDOS plots will be built and the quantum capacitance will be estimated. On the basis of the obtained data, some of the features of the interaction between the LTO and carbon nanostructures that were observed in the experiments are explained. A comparison of CNT and graphene of different concentrations will reveal the most appropriate material for combination with LTO considering their application in LIBs and SCs.

## 2. Methods

All calculations were performed by an ab initio method realized in the SIESTA 4.1.5 software package [24]. Exchange and correlation potentials were described by generalized gradient approximation (GGA) of the Perdew–Burke–Ernzerhof (PBE) [25] function with the application of the Grimme correction to take into account the van der Waals interaction [26]. This approach is also known as PBE-D2. A double-zeta polarized (DZP) orbital basis set was applied for Li and Ti atoms, while the facilitated double-zeta (DZ) potential was applied for the O and C atoms. The polarized orbitals for C and O atoms accounted for only 4 and 0.7%, respectively, of the total charge, which was about 25% for Li and Ti. So, we chose the DZ potential for C and O atoms for time saving. The Hubbard correction (*U_eff_*) including the effective magnetic exchange interaction parameter and the on-site Coulomb interaction was applied to describe the strong correlated effect of electrons in the Ti-3d states [27]. In our calculations, we set *U_eff_* = 5 eV in order to obtain the desired value of the LTO band structure. The models were fully relaxed until the forces acting on the atoms were smaller than 0.04 eV/Å, and the kinetic energy cut-off was set to 300 Ry. A Monkhorst–Pack grid of 8 × 8 × 1 was applied to calculate the total energy of the 2D films, while a grid of 8×8×8 was used for the 3D LTO. The k-point mesh was sampled by 24 × 24 × 1 for the total and partial density of states (DOS and pDOS) in the case of the 2D films. For 3D LTO, a 24 × 24 × 24 k-point grid was applied. For the DOS calculation, Gaussian smearing with a small broadening width of 0.05 eV was used.

The formation energy $E_{B}$ between the carbon nanostructures and LTO film was calculated by the following formula:

(1) $$E_{B}=E_{comp}-E_{C}-E(LTO),$$

where *E_comp_* is the energy of the composite, *E_C_* is the energy of the isolated carbon nanomaterial, and *E(LTO)* is the energy of the isolated LTO film.

The quantum capacitance (QC) was first defined by Luriy as the difference between a charge with an alteration in the Fermi level [28]. For low-dimensional structures, QC resulted in a more significant additive to the total capacitance in comparison with the geometric one. Thus, its calculation is essential for materials that can be applied in LIBs. We applied the following formula to obtain QC ($C_{Q}$) [29,30]:

(2) $$C_{Q}=\frac{1}{mV}\int_{0}^{V}eD(E_{F}-eV^{\prime })dV^{\prime }$$

where *m* is the mass structure, *V* is the applied voltage that corresponds to a shift in Fermi level *E_F_*, D is the area under the plot of density of state (DOS) in a considered range, and e is the elementary charge. The applied voltage was applied in steps equal to 0.05 eV. To find the energy pathway of the lithium atoms between the two local minima in the LTO and graphene/LTO structures, the Nudge Elastic Band (NEB) [31] approach was used.

## 3. Results

### 3.1. Construction of LTO Atomistic Models

**Figure 1:**
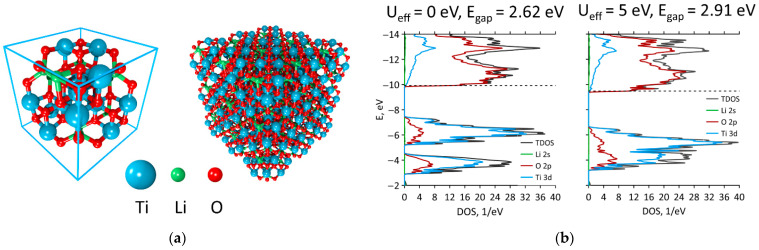
Three-dimensional Li_4_Ti_5_O_12_: (**a**) the unit cell and the bulk structure; (**b**) DOS and pDOS without adding the Hubbard parameter (**left**) and with its addition (**right**).

To obtain the unit cell of Li_4_Ti_5_O_12_, we substituted three titanium atoms with three lithium atoms in the unit cell of Li_1.333_O_4_Ti_1.667_ with an *Fd3m* space group [32] (Figure 1a). It is known that the choice of substituted titanium atoms significantly affects the energy gap of LTO. In our case, we aimed to achieve an energy gap of 2.95 eV as observed in the experiment [8]. Minimization of energy by the atomic coordinates showed optimal lattice vectors equal to 8.640, 8.623, and 8.638 Å, respectively, which were close to previous ab initio studies [20,23].

The DOS of the 3D LTO without the addition of the Hubbard parameter demonstrated an energy gap of 2.62 eV (Figure 1b). The addition of the Hubbard parameter *U_eff_* enlarged the gap to 2.91 eV, which corresponded to the above-mentioned experimental value. An analysis of pDOS showed that the valence band was mainly determined by O 2p states (Figure 1b). The Fermi level (–9.48 eV) was found at the end of the valence band. As the energy gap was raised from 2.62 to 2.91 eV, the position of the Fermi level shifted. The conduction band was mainly comprised of Ti 3d states, which confirmed the previous modeling results [23].

**Figure 2:**
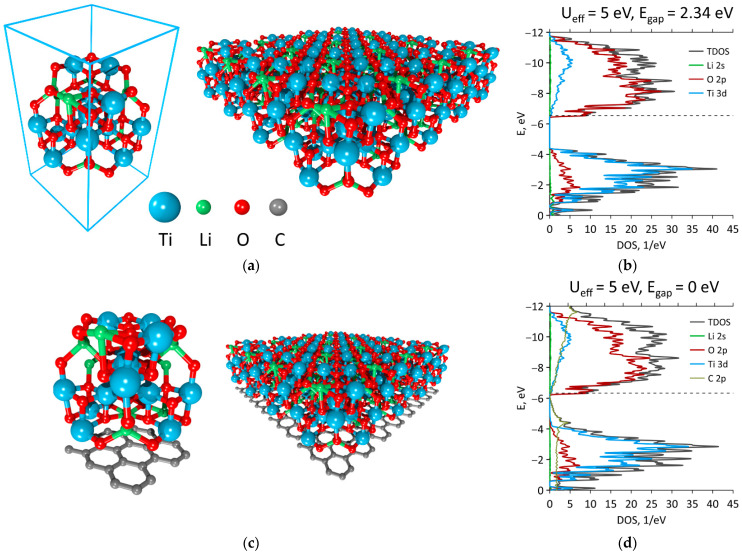
Two-dimensional LTO and G/LTO films: (**a**) unit cell and translated supercell of LTO, (**b**) DOS and pDOS of LTO, (**c**) supercell and translated supercell of G/LTO, and (**d**) DOS and pDOS of G/LTO.

For the 2D LTO film model, the lattice vector along the z axis was set to 300 Å to exclude the interaction between images after translation (Figure 2a). After the lattices were optimized, two other translation vectors were equal to 8.36 Å. The Fermi level shifted to −6.54 eV, while the energy gap decreased to 2.62 eV, but remained at the end of the valence band (Figure 2b). The domination of O 2p states in the valence band and Ti 3d states in the conduction band was also observed, as in case of the 3D LTO.

### 3.2. Graphene + LTO

The unit cell of one layered graphene consisted of 24 carbon atoms. The optimal translation vectors of graphene in this case were 7.38 Å along the zigzag edge and 8.52 Å along the armchair edge. The C-C bond lengths of ideal graphene were equal to 1.419 and 1.421 Å, which corresponded well with previous studies [33,34]. The optimal translation vectors for the G/LTO film were 7.68 Å along the zigzag edge and 8.70 Å along the armchair edge. So, the graphene layer stretched 4.07% and 1.02% along the zigzag and armchair edges, respectively. The C-C bonds in graphene changed to 1.414 Å and 1.549 Å, which confirmed stretching. One of the LTO vectors was compressed by 8.8%, while another was stretched by 4.15% in comparison with 2D LTO. Thus, to match the lattice vectors of LTO, graphene layers were initially strained by 17% along the zigzag edge (Figure 2c). The DOS and pDOS of G/LTO with one graphene layer are shown in Figure 2d. It can clearly be seen that the contribution of graphene’s carbon 2p states destroyed the energy gap and DOS demonstrated the conductor properties. It can also be seen that the peaks of C atoms did not match the peaks of LTO atoms, indicating the absence of hybridization between the composite’s components. Thus, the main type of interaction between LTO and graphene was determined by the van der Waals forces.

**Figure 3:**
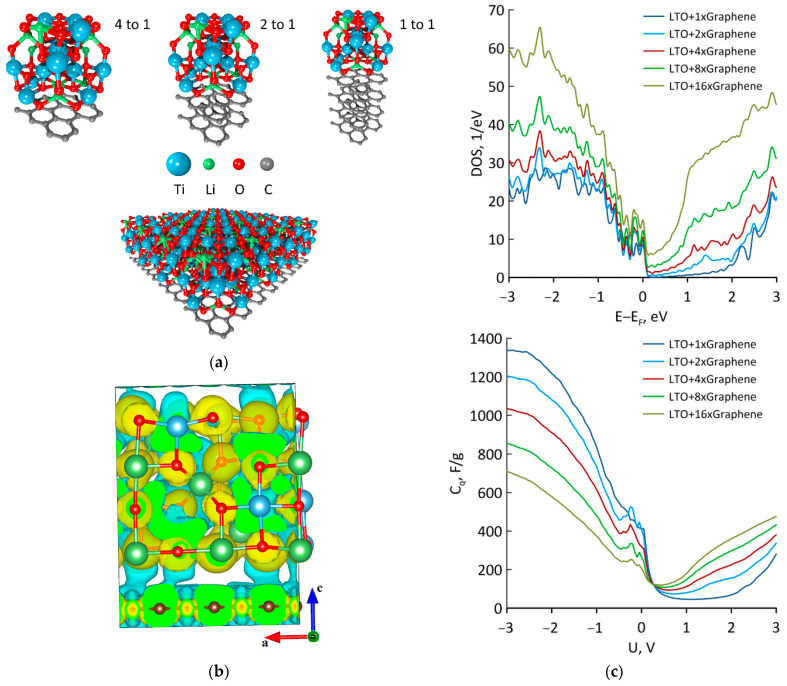
G/LTO nanocomposites: (**a**) the supercells of G/LTO with m(LTO):m(G) 4:1; 2:1 and 1:1 and the expanded 4:1 supercell; (**b**) volumetric data for charge distributions or wave functions; (**c**) DOS and QC plots.

The different mass ratio between the graphene and LTO in the composite was regulated by the number of graphene layers (Figure 3a). The mass of four graphene layers was equal to the mass of the one LTO unit cell. In all cases, the process of G/LTO composite formation was exothermic and about 1.53–1.71 eV of energy was released (Table 1). The distance between the graphene and LTO was in the range of 2.732–2.754 Å and no chemical bonds between the components were observed. An increase in graphene concentration led to a rise in Fermi level, which was also observed in the experiments [14]. After composite formation, the LTO cell transferred part of the charge to the graphene mesh. The largest amount of charge was recorded in the cases of mass ratio m(LTO):m(G), which was equal to 4:1 and 1:2 (Table 1). An analysis of the charge distribution difference showed that the most noticeable interaction occurred between the graphene and Ti atoms of LTO (Figure 3b).

The analysis of DOS showed that an increase in graphene concentration significantly strengthened the conductive properties of the material (Figure 3c). For the 2D LTO film, the DOS value at the Fermi level was equal to 9.20 eV^−1^. When the mass ratio m(LTO):m(G) was equal to 1:1, this value increased to 10.44 eV^−1^. As shown in the comparison in Table 1, the DOS value increased to 12.27 eV^−1^ and 15.05 eV^−1^ when the mass ratios were equal to 1:2 and 1:4, respectively. The situation was opposite when analyzing the QC values. The maximum QC value at 0 V was about 668.11 F/g for 3D LTO, which decreased to 583.19 F/g for the 2D film. After increasing the graphene concentration, the maximum QC value steadily dropped and reached 205.50 F/g when the mass of graphene was four times higher than the mass of LTO (Table 1). It can be concluded that enlarging the graphene concentration in the G/LTO composite led to enhanced conductivity, while enlarging LTO caused an increase in QC following the predominance of the non-Faradaic process during the charge–discharge cycles. When the voltage dropped from 0 to −3 V, the QC significantly rose (left part of the QC plot in Figure 3c). In comparison, when the voltage was raised from 0 to 3 V, the change in QC was insignificant (right part). The process of voltage decrease corresponded to the discharge (lithiation) process, while the process of voltage increase was related to the charge (delithiation) process. Therefore, it can be concluded that in real experiments, the non-Faradaic process prevailed during the discharge cycle and Faradaic process prevailed during the charge cycle. The noticeable peak of the QC curve at −0.2 V may indicate the beginning of possible chemical reactions.

### 3.3. CNT + LTO

**Figure 4:**
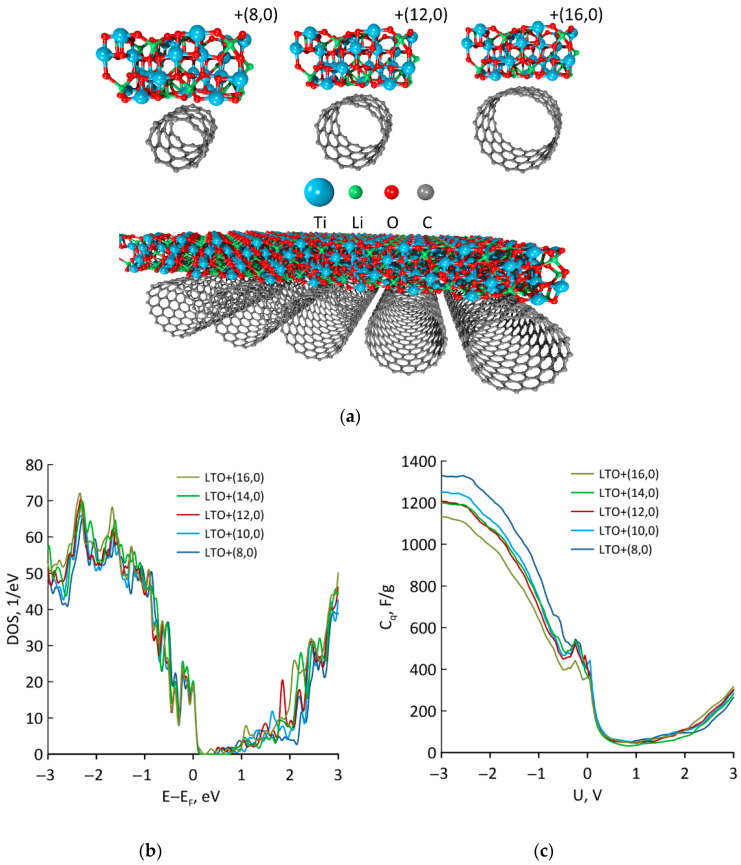
CNT/LTO nanocomposites: (**a**) atomic supercells of CNT(8,0)/LTO, CNT(12,0)/LTO, CNT(16,0)/LTO and expanded supercell of CNT(16,0)/LTO; (**b**) DOS; and (**c**) QC plots.

In our paper, we considered the interaction of LTO with CNTs (16,0), (14,0) (12,0) (10,0), and (8,0). The supercells of CNT/LTO composites are shown in Figure 4. This type of package was observed in the experiments in [19]. Herein, the intertube distance varied from 10.13 Å in the case of CNTs (8,0) to 3.76 Å in the case of CNTs (16,0). An intertube distance of 3.42 Å was observed in [19], so CNT (16,0)/LTO was the closest to the experiment composite. Herewith, the further the tube’s radius, the greater the distance between CNT and LTO (Table 2). The formation energy increased with the growth of CNT’s radius (Table 2), which could be explained by the growth in interacting atoms between LTO and CNT. So, the application of tubes with a higher diameter was more energetically favorable for the synthesis of CNT/LTO composites. Analyzing this trend, we could suppose that for tubes with a diameter greater than 29.6 Å, the CNT/LTO composites would be stronger than G/LTO. It can be seen that CNT accepted a charge that was two times higher than for graphene, as CNT’s atoms were more chemically active than graphene because of its geometry. The amount of this charge did not depend on the CNT radius. As for graphene, the presence of CNT significantly decreased the Fermi level of LTO (Table 2). As seen from Table 2, the Fermi level of the pristine CNT (8,0) was −4.66 eV and for the CNTs (10,0), (12,0), (14,0), and (16,0), the Fermi level was almost the same (−4.40 eV). So, the Fermi level of the of the obtained CNT/LTO samples almost did not depend on the CNT’s radius and lay in the range of −6.07 ≤ *E_F_* ≤ −5.85 eV. As in the case of graphene, the addition of CNT raised the DOS value at the Fermi level and decreased QC at 0 V. The obtained results confirm the experiments data of Ye et al., who observed a reduction in the specific capacity of CNT/LTO with the increase in the amount of CNT [19]. Such a reduction was mainly caused by decreasing the quantum capacitance in the composite. The behavior of the QC curve for the CNT/LTO composites repeated the behavior of the QC curve for G/LTO.

Let us compare the QC plot of G/LTO and CNT/LTO for similar ratios between the carbon and LTO atoms. The mass ratio of 2:1 was almost similar for G/LTO (precise mass ratio 2.14:1) and for CNT(12,0)/LTO (precise mass ratio 2.14:1). The QC for both cases is shown in Figure S1 for convenience. It can clearly be seen that QC was almost similar. So, at the same concentration, the interaction between LTO and carbon materials such as graphene and CNT caused the same QC.

## 4. Conclusions

Molecular modeling has proven itself an effective tool for exploring the materials used in the chemical power sources. In this work, the supercells of G/LTO and CNT/LTO nanocomposites, often used as anode materials for LIBs and SCs, were investigated for the first time. Graphene demonstrated high conductive properties as its band structures did not demonstrate an energy gap at the Fermi level. Opposite to graphene, 2D LTO had an energy gap of *E*_gap_ = 2.34 eV. The addition of graphene to LTO reduced the energy gap and pDOS evidently, signifying the contribution from C atoms. The interaction between graphene and LTO was dominated by long-range van der Waals interactions, and the most noticeable overlap of atomic orbitals was observed between the C and Ti atoms. According to the QC of pure LTO (which aligned with previous experimental data), increasing either the graphene or CNT concentration in the supercell led to a decrease in QC. In particular, the magnitude of QC depended on the C ratio, but did not depend on the type of carbon nanomaterials. The QC of graphene at the Fermi level was close to zero, which was about 583.19 F/g for 2D LTO. In other words, a higher concentration of LTO yielded a larger QC magnitude. As QC corresponded to the non-Faradaic process, which is associated with the accumulation of charge, increasing the LTO concentration would make the non-faradaic process more dominant. According to the relationship between QC and voltage in the real experiments, the non-Faradaic process prevailed during the discharge cycle (lithiation) and the Faradaic process prevailed during the charge cycle (delithiation).

## Figures and Tables

**Table 1:** Electronic and energetic properties of G/LTO composites with different mass ratios.

Structure	Approximate Mass Ration m(LTO): m(G) (Precise Mass Ratio)	Formation Energy, eV	Fermi Level (Fermi Level of Pure Graphene), eV	Graphene-LTO Distance, Å	Transferred Charge in Graphene, e	DOS at the Fermi Level, eV^−1^	QC at 0 V, F/g
2D LTO	-	-	−6.54	-	-	9.20	583.19
3D LTO	-	-	−9.48	-	-	11.02	668.11
LTO + 1 × Graphene	4:1 (4.28:1)	−1.53	−6.33 (−4.54)	2.754	0.134	8.60	413.88
LTO + 2 × Graphene	2:1 (2.14:1)	−1.66	−6.23 (−4.60)	2.74	0.083	9.60	408.27
LTO + 4 × Graphene	1:1 (1.07:1)	−1.69	−6.22 (−4.65)	2.738	0.088	10.44	319.32
LTO + 8 × Graphene	1:2 (1:1.87)	−1.71	−6.14 (−4.68)	2.752	0.115	12.27	263.47
LTO + 16 × Graphene	1:4 (1: 3.74)	−1.65	−5.75 (−4.75)	2.732	0.051	15.05	205.50

**Table 2:** Electronic and energetic properties of the CNT/LTO composite with different mass ratios.

Structure	Mass Ratio m(LTO): m(CNT)	Formation Energy, eV	Fermi Level, eV (Fermi Energy of the Pure CNT)	Distance between Tubes and LTO, Å	Transferred Charge on the Tube, e	DOS at the FERMI Level, eV^−1^	QC at 0 V, F/g
2D LTO × 2	-	-	−6.54	-	-	18.40	583.19
LTO + (8,0)	3.21:1	−1.39	−6.06 (−4.66)	2.479	0.225	18.22	400.25
LTO + (10,0)	2.57:1	−1.34	−5.95 (−4.40)	2.563	0.219	19.24	424.38
LTO + (12,0)	2.14:1	−1.53	−5.90 (−4.37)	2.585	0.262	19.68	407.35
LTO + (14,0)	1.83:1	−1.79	−5.99 (−4.39)	2.555	0.254	20.23	369.04
LTO + (16,0)	1.60:1	−1.92	−5.85 (−4.38)	2.629	0.279	19.12	360.00

## Data Availability

Not applicable.

## Supplementary Materials

The following supporting information can be downloaded at: https://www.mdpi.com/article/10.3390/ma16083270/s1. Figure S1: DOS and QC plots for G/LTO with mass ratio 2:1 (precise mass ratio 2.14:1) and for CNT(12,0)/LTO (precise mass ratio 2.14:1). Figure S2: Volumetric data for charge distributions or wave-functions for CNT (8,0)/LTO and CNT (16,0)/LTO.
