# Supplementary material for: Two-Dimensional Films Based on Graphene/Li4Ti5O12 and Carbon Nanotube/Li4Ti5O12 Nanocomposites as a Prospective Material for Lithium-Ion Batteries: Insight from Ab Initio Modeling
Source: Materials (Basel). 2023 Apr 21;16(8):3270. doi: 10.3390/ma16083270 (PMC10146994; doi:10.3390/ma16083270)
Supplement: Supplementary file 1 [file materials-16-03270-s001.zip › materials-2324525-supplementary.pdf]

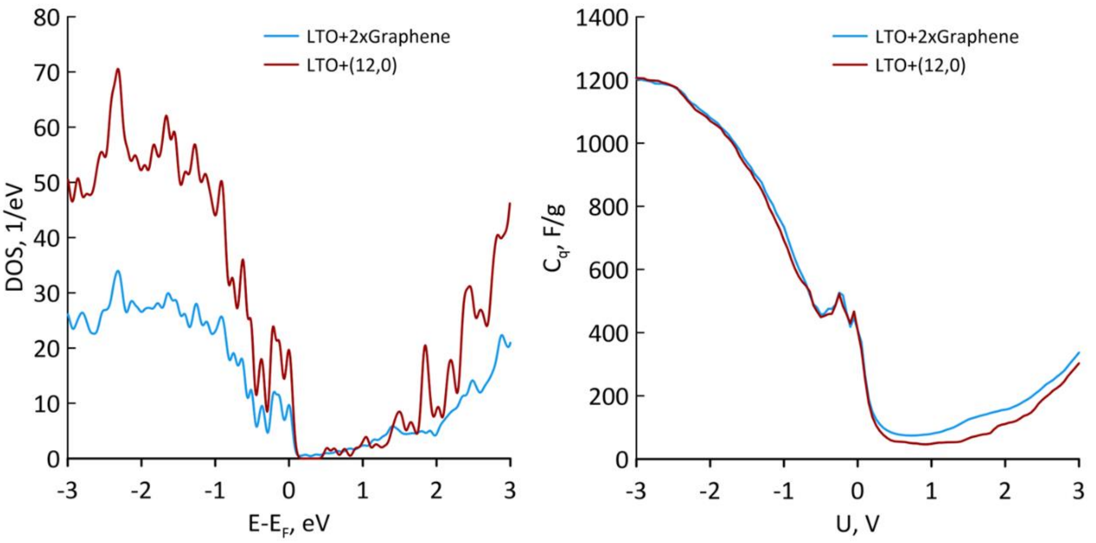


Figure S1. DOS and QC plots for G/LTO with mass ratio 2:1 (precise mass ratio 2.14:1) and for CNT(12,0)/LTO (precise mass ratio 2.14:1).

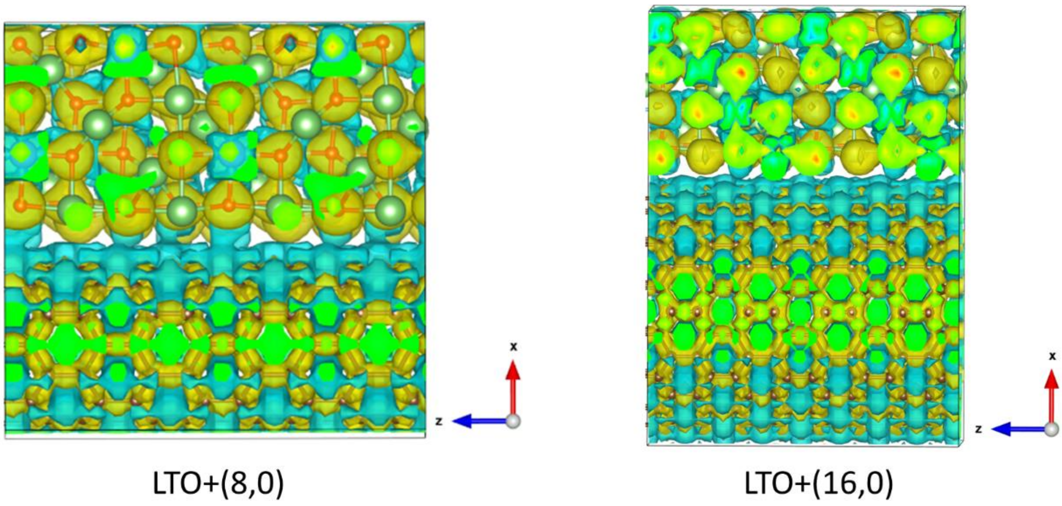


Figure S2. Volumetric data for charge distributions or wave-functions for CNT (8,0)/LTO and CNT (16,0)/LTO .
